# Insertion of N_2_ into the Channels of AFI Zeolite under High Pressure

**DOI:** 10.1038/srep13234

**Published:** 2015-08-18

**Authors:** Hang Lv, Mingguang yao, Quanjun Li, Ran Liu, Bo Liu, Zhen Yao, Dedi Liu, Zhaodong Liu, Jing Liu, Zhiqiang Chen, Bo Zou, Tian Cui, Bingbing Liu

**Affiliations:** 1 State Key Laboratory of Superhard Materials, Jilin University, Changchun 130012, P. R. China; 2 Institute of New Energy, Bohai University, Jinzhou, Liaoning, 121000, China; 3 Beijing Synchrotron Radiation Facility, Institute of High Energy Physics, Chinese Academy of Sciences, Beijing 100049, China; 4 GeoScience Department, Stony Brook University, Stony Brook, New York 11794, United States

## Abstract

We present an experimental study of a new hybrid material where nitrogen is encapsulated in the channels of porous zeolite AlPO_4_-5 (AFI) single crystals by a high-pressure method. The high-pressure behavior of nitrogen confined inside the AFI nano-channels is then investigated by Raman spectroscopy up to 44 GPa. Under pressure, the Raman modes of confined nitrogen show behaviors different from those of the bulk nitrogen. After the return to atmospheric pressure, it is demonstrated that non-gaseous nitrogen can be effectively stabilized by being confined inside the intact AFI sample. This result provides new insight into nitrogen capture and storage technologies.

Among the diatomic molecules, nitrogen has a special place since it has the greatest binding energy and, except for H_2_, the shortest bond length^1^,^2^. Experimental and theoretical work has revealed a complicated phase diagram for nitrogen^3^,^4^. Under high pressure there are several phase transitions in nitrogen which change the structure into molecular phases, non-molecular phases and a polymeric phase. In the past few years, the cubic gauche phase has been found under high pressure and temperature, in which the nitrogen atoms connect with single N-N bonds into a polymeric network. It is the highest-energy pure nitrogen material known and releases a large amount of energy under the transformation to the molecular state. The energy released is much larger than that of the most powerful energetic materials^4^,^5^,^6^. Based on these facts, the study of nitrogen under high pressure not only attracts much attention as a fundamental problem of chemistry, but may also shed light on the principles of structural transitions in diatomic molecular systems. In recent years, studies of nitrogen confined by some host materials have been initiated. Such studies can contribute to discovering new physical phenomena and reveal the physical properties of the diatomic molecular system. It was theoretically predicted that atomic chains could be effectively stabilized by being confined in carbon nanotubes, silicon carbide nanotubes or in a multilayer graphene matrix^7^,^8^,^9^,^10^. However, it is still unclear what behavior of nitrogen will actually be induced by the confinement in some template. In particular, it is still unknown whether or not the nitrogen will choose to conform to the known high-pressure behavior under confinement.

Like carbon, zeolite is an ideal template to study the confinement effect^11^. It has been demonstrated that under pressure and elevated temperatures CO_2_ can be inserted into the small-pore zeolite natrolite^12^. In the zeolite family, AlPO_4_-5(AFI), a type of transparent microporous crystal containing one-dimensional channels with a suitable size of 0.73 nm, has been used for introducing some guest molecular species, such as selenium and iodine molecular chains, into the nano-pores^13^,^14^,^15^. Besides, the sorption capacity of nitrogen molecules (size 0.354 nm) in AFI has been investigated in earlier studies, related to the channel structures of AFI^16^,^17^. The above facts put together motivate us to investigate whether the porous zeolite AFI can become a storage medium for the high pressure phases of nitrogen at ambient pressure and room temperature, and to use the high-pressure method to study the stability of nitrogen confined in the channels of AFI. This can lead to a much improved understanding of the moleculare structure of nitrogen and give an objective basis for the investigation of diatomic molecular systems. In this paper we focus on the incorporation of nitrogen into the channels of AFI crystals by a high-pressure method. We also report the high-pressure stability of nitrogen confined inside the AFI channels using *in situ* Raman spectroscopy measurements under room temperature. Interestingly, the Raman modes of confined nitrogen show behaviors different from those of the bulk nitrogen. After releasing pressure, it is experimentally verified by Raman and EDX spectroscopic study that non-gaseous nitrogen can be effectively stabilized when encapsulated in the channels of intact AFI sample.

## Results

**Figure 1:**
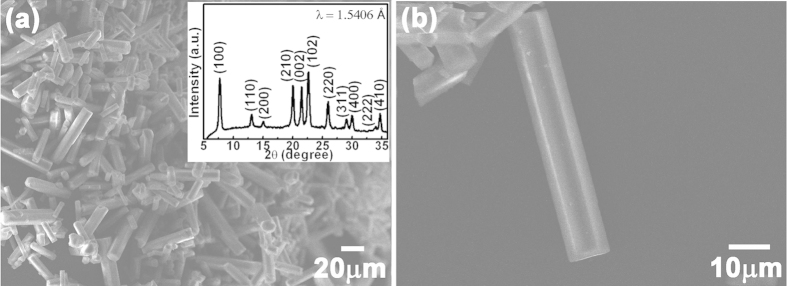
SEM images of the AFI crystals. The inset shows the corresponding XRD spectrum.

The morphologies of the as-synthesized AFI crystals observed by SEM are shown in Fig. 1. SEM images reveal that the crystals of AFI have a typical hexagonal rod-like morphology with dimensions of 20 μm–100 μm in length and 5 μm–10 μm in cross section diameter. The XRD pattern of AFI crystals at ambient conditions is shown in the inset of Fig. 1(a). All peaks can be indexed to hexagonal AlPO_4_-5. The AFI framework is constructed from alternating tetrahedra of (AlO_4_)^−^ and (PO_4_)^+^, forming parallel open channels with an inner diameter of about 0.73 nm, which is an ideal template to allow the nitrogen molecules with size of about 0.354 nm to be intercalated in the channels.

**Figure 2:**
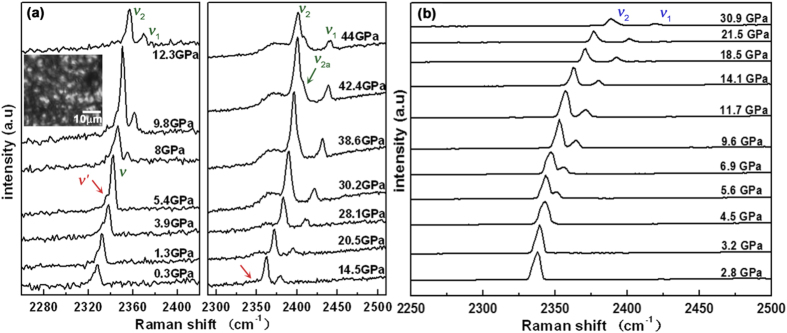
High-pressure Raman spectra of (**a**) nitrogen in the confined system and (**b**) bulk nitrogen. The inset shows an optical image of the AFI sample in the confined system at 22.8 GPa.

Raman spectra of the N_2_ vibrons observed in our confined system at various pressures are shown in Fig. 2(a). In the spectra below 5.4 GPa, the main peak ν around 2335 cm^−1^ belongs to both the liquid nitrogen (≤1.3 GPa) and the *β*-N_2_ (>1.3 GPa), for which the vibrational frequency has symmetry *A*_1g_^18^. Besides that, a small shoulder *ν′* on the low-frequency side of the peak can be noticed. Compared with the Raman spectra of bulk nitrogen shown in Fig. 2(b), it is suggested that the extra shoulder *ν′* originates from the nitrogen confined in the AFI sample. At above 5.4 GPa, accompanying the transition from *β*-N_2_ to *δ*-N_2_, the ν peak splits into two peaks ν_1_ and ν_2_. Same splitting is also observed in bulk nitrogen^19^,^20^,^21^. At pressures above 16 GPa, a phase transition from *δ*-N_2_ to *ε*-N_2_ is indicated by x-ray diffraction studies^3^. In the transition range the vibronic regime shows no changes in the spectral features. Only at 42.4 GPa a small shoulder appears on the high-frequency side of the ν_2_ mode (ν_2a_), just as has been reported in bulk nitrogen^19^,^22^.

We fit the Raman vibrational modes by Lorentz functions [see Figure S1] and obtained the pressure dependences of the Raman spectra [see Figure S2]. As we can see from the Figure S1, a small shoulder ν*′* is clearly detectable on the low-frequency side from 1.3 GPa to 14.5 GPa. With increasing pressure the ν_1_ and ν_2_ vibrations shift to higher frequencies, the shoulder ν*′* could be conspicuously distinguished from ν_2_ vibration at pressures above 14.5 GPa, and the broad peak (ν*′*) appeared at around 2350 cm^−1^ since then. Pressure dependences of the Raman spectra [Figure S2 (a)] suggest that the ν, ν_1_ and ν_2_ vibrations probably originate in the nitrogen outside the AFI channels. In low-pressure areas, the vibrational mode ν shows an asymmetric broadening, which could be explained by some nitrogen being squeezed into the AFI channels, where the pressure might be a little lower than outside. The small shoulder ν*′* probably corresponds to nitrogen inside the channels. It is suggested that the vibrational mode of nitrogen splits, indicating two different conformations of the nitrogen molecules, i.e., the one (ν_1_ and ν_2_ vibrations) in the pressure medium and the other (the broad peak on the low-frequency side of ν_2_) inside the AFI channels. A similar Raman mode splitting under high pressure by the confinement in some porous zeolite has been found in CO_2_^12^. Due to the lower nitrogen content inside the AFI channels than outside, the broad peak is lower in intensity.

Preliminary studies in our group have suggested that the AFI structure is distorted in the pressure range 4.8 GPa to 15.9 GPa. At above 15.9 GPa, all diffraction peaks disappeared, suggesting that the AFI crystals were seriously distorted^23^. The inset optical image in Fig. 2(a) clearly shows the distorted AFI at 22.8 GPa. Only a few AFI crystals keep their rod-like morphology, and the fragmented blocks seen in the DAC are made up from pieces of AFI with sizes of 1 μm–5 μm. That means the long-range order of the one-dimensional channels is destroyed and replaced by localized short-range structures. In the local regions with a high nitrogen concentration, it is suggested that the confined nitrogen molecules have been squeezed toward each other and the wall, and the confined nitrogen transformed to an amorphous structure. The broad peak on the low-frequency side of ν_2_ in our Raman spectra most probably arises from the amorphous nitrogen confined by the seriously distorted AFI channel. In high-pressure studies of other molecular crystals, such as C_60_/C_70_ powder mixtures, the Raman spectrum has been clearly identified as that of amorphous carbon, with broad peaks under pressure^24^.

By fitting the measured pressure dependence of the Raman shift [Figure S2 (b)], we obtain the corresponding pressure coefficients for bulk nitrogen (ν and ν_2_-the main peak of high-pressure phases) and confined nitrogen (ν*′*), as shown in Table 1. Except in the range 0.2–2.4 GPa, the pressure coefficients of the confined nitrogen are closed to those for bulk nitrogen. This indicates that the pressure-induced changes in the vibrational mode of confined nitrogen molecules are similar to those in bulk nitrogen, but due to the confinement inside AFI channels the pressure coefficients are a little lower. That probably means the interaction among confined nitrogen molecules and channel molecules is similar to the interaction among bulk nitrogen molecules. In the pressure range of 0.2–2.4 GPa, the pressure coefficient is found to be 3.3 for the confined nitrogen, which is much larger than the value 2.5 for bulk liquid N_2_ and close to the value 3.1 found for bulk *β*-N_2_. It is reasonable to suppose that in the local regions with a higher nitrogen concentration the nitrogen molecules are squeezed into AFI channels, and under the confinement in the channel the intermolecular interaction of nitrogen are strengthened. This is similar to what happens in bulk *β*-N_2_. By this token, the nitrogen molecules in the AFI channels probably originally form a solid, and when AFI crystals were seriously distorted above 15.9 GPa the interaction between confined nitrogen molecules and channel molecules is similar to the interaction among bulk nitrogen molecules.

**Figure 3:**
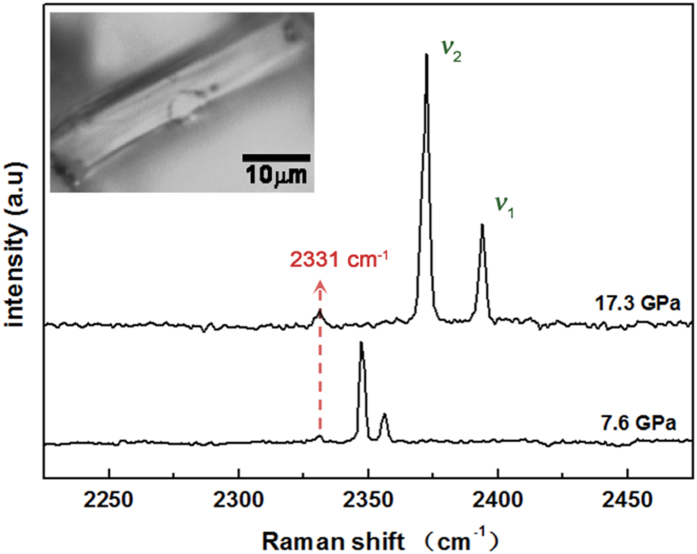
High-pressure Raman spectra of nitrogen in the intact AFI confined system. The inset shows an optical image of the intact AFI sample in the diamond anvil cell.

To characterize the nitrogen confined in the channels of the intact AFI crystals under high pressure, we loaded a small amount of AFI crystals into the sample chamber and attempted to adjust their locations to make sure that they exerted little pressure on each other in experiment. Thus the hydrostatic conditions were improved and the AFI crystals kept an intact shape under pressure (see the inset optical image in Fig. 3). High-pressure Raman spectra of nitrogen in the intact AFI confined system are shown in Fig. 3. Compared with the Raman spectra of bulk nitrogen at room temperature in Fig. 2(b), besides the ν_1_ and ν_2_ vibrations peaks, there is an additional small peak at 2331 cm^−1^. The small peak could be attributed to the non-gaseous nitrogen confined in the channels of AFI in local regions with a low concentration, since a similar nitrogen peak appears in the spectrum of AFI channels upon decompression to ambient pressure (see the discussion below). It is interesting that the small peak at this low frequency did not shift to higher frequency with increasing pressure, which means pressure was very low inside the intact AFI channels and the nitrogen molecules in the channels were protected from any influence of the pressure from 7.6 GPa to 17.3 GPa. Therefore, it is reasonable that the supporting effect of the AFI framework plays a role in the structural transition of nitrogen inside the AFI channels under pressure. The phase transition in bulk nitrogen does not occur in nitrogen confined in the intact AFI sample at room temperature and high pressure.

**Figure 4:**
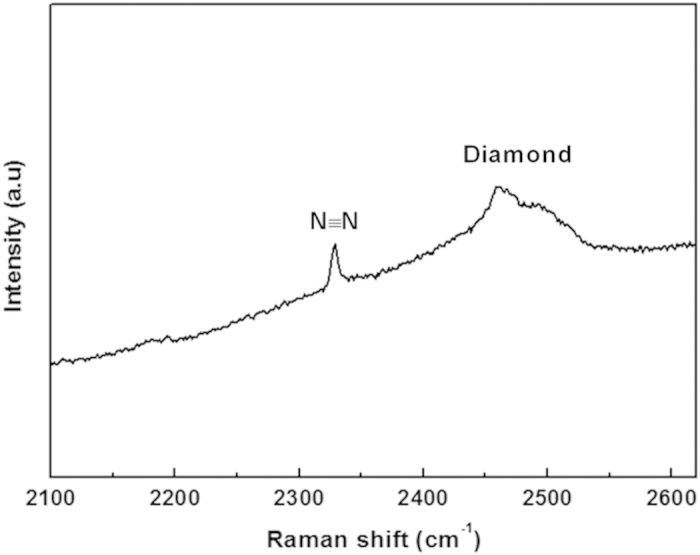
Raman spectrum of the sample: nitrogen confined inside the AFI channels after releasing the pressure to 0 GPa .

After decompression to ambient pressure, it is important to note that there are differences between Raman spectra from the nitrogen confined in seriously distorted AFI and in intact AFI. No peak from nitrogen was present in the spectrum of the seriously distorted AFI after pressure release. The Raman spectrum of the intact AFI sample at ambient conditions after releasing the pressure from 33 GPa is shown in Fig. 4. A single prominent peak from molecular nitrogen appears at about 2328.5 cm^−1^. EDX spectrum of the intact AFI sample [Figure S3] also shows a small N signal. The XRD pattern of the intact AFI shows only a very broad peak [Figure S4]. It means the AFI sample released from high pressure shows an intact shape, yet the internal structure is amorphous. All of these results clearly demonstrate a well-defined compositional profile of nitrogen confined in our AFI sample.

**Figure 5:**
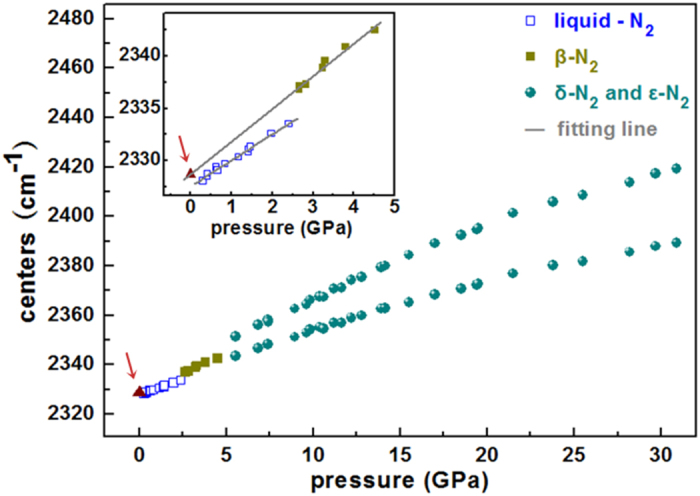
Pressure dependence of Raman spectra of nitrogen confined in AFI and bulk nitrogen. The red arrow marks the nitrogen confined in AFI at 0 GPa.

To further characterize the confined nitrogen, the results for the Raman frequency shifts in bulk nitrogen and the nitrogen confined in AFI sample after pressure release are given in Fig. 5. The triangle (arrow) shows data for confined nitrogen and the frequency shifts for bulk nitrogen are shown by the squares and circles^25^. The data point for the nitrogen confined in AFI lies on the fitted line for *β*-N_2_, which probably means that the nitrogen confined in AFI maintains the structure of *β*-N_2_. Another possibility is that liquid nitrogen is confined in the AFI zeolite, and there is a pressure difference between the inside and outside of the AFI. If the pressure inside the AFI is 0.4 GPa, in pure fluid nitrogen, the Raman frequency is 2328.5 cm^−1^ at 0.4 GPa, and the pressure outside AFI is 0 GPa after pressure release. From these Raman and EDX spectra we can only infer that the confined nitrogen is either in the liquid phase or in the solid *β*-phase. Further studies by other techniques are necessary to get more detailed information about the structural transition of the nitrogen confined in the AFI crystals, for example, to determine the atomic conformation of nitrogen inside the one-dimensional channels of AFI. From the Raman spectrum [Figure S5] of nitrogen confined in the intact AFI sample after preservation for a long time at atmosphere pressure and room temperature, we conclude that non-gaseous nitrogen could be effectively stabilized for at least 125 days, and the content of confined nitrogen tended to decrease as time went on.

## Conclusions

We have demonstrated that under pressure non-gaseous nitrogen can be inserted into the channels of AFI single crystal. The high-pressure behavior of the nitrogen confined inside the AFI channels is investigated by Raman spectroscopy. Different of bulk nitrogen, at high nitrogen concentrations, the nano-confined N≡N mode Raman peak evolved with increasing pressure from splitting to broadening, and at low nitrogen concentrations, both the peak position and shape remained unchanged with varying pressures. We also realize the porous zeolite AFI can become a storage medium for the high pressure phases of nitrogen at atmospheric pressure and room temperature. A detailed description of the structure of nitrogen confined within the channels must be established in the future. Chemical and structural modifications of the AFI structure can be made in order to modify the insertion conditions and allow its use in nitrogen capture and storage technologies.

## Methods

The AFI sample was synthesized by a simple method as described in Ref. 26. The pressure was generated in a diamond anvil cell (DAC) with a culet size of 400 μm. AFI together with the liquid quasihydrostatic pressure-transmitting medium silicon oil were loaded into a 120 μm hole drilled in a T301 stainless steel gasket. A ruby with a diameter of about 5 μm was put in the sample chamber and the pressure was determined using a ruby fluorescence method. Nitrogen was loaded by immersing the DAC in liquid nitrogen, and the cell was pressurized to a few GPa.

High-pressure Raman spectra were recorded on a Renishaw inVia Raman Microscope in the backscattering geometry using an argon ion laser with the 514.5 nm line, provided with a CCD detector system. Raman modes were analyzed by fitting the spectra to Lorentzian functions to determine the line shape parameters. The morphology and composition of the products were characterized using the scanning SEM (JSM-6480LV) equipped with electron energy-dispersive X-ray (EDX).

## Additional Information

**How to cite this article**: Lv, H. *et al.* Insertion of N_2_ into the Channels of AFI Zeolite under High Pressure. *Sci. Rep.* **5**, 13234; doi: 10.1038/srep13234 (2015).

## Supplementary Material

Supplementary Information

## Figures and Tables

**Table 1:** Pressure coefficients of Raman shift for confined nitrogen and bulk nitrogen.

**Pressure (GPa)**	**Pressure coefficient of confined nitrogen (**ν**΄)**	**Pressure coefficient of bulk nitrogen (**ν **and** ν_**2**_)
0.2–2.4	3.3	2.5 (liquid-N_2_)
2.6–5	2.9	3.1 (*β*-N_2_)
5.4–12.9	1.9	2.2 (*δ*-N_2_)
15.5–33	1.1	1.5 (*ε*-N_2_)
