# Supplementary material for: Insertion of N2 into the Channels of AFI Zeolite under High Pressure
Source: Sci Rep. 2015 Aug 18;5:13234. doi: 10.1038/srep13234 (PMC4539611; doi:10.1038/srep13234)
Supplement: Supplementary Information [file srep13234-s1.pdf]

Supporting Information

Insertion of N_2 into the Channels of AFI Zeolite under High Pressure

Hang Lv,[1,2] Mingguang yao,[1] Quanjun Li,[1] Ran Liu,[1] Bo Liu,[1] Zhen Yao,[1] Dedi Liu,[1] Zhaodong Liu,[1] Jing Liu,[3] Zhiqiang Chen,[4] Bo Zou,[1] Tian Cui,[1] and Bingbing Liu[1*]

[1]State Key Laboratory of Superhard Materials, Jilin University, Changchun 130012, P. R. China

[2]Institute of New Energy, Bohai University, Jinzhou, Liaoning, 121000, China

[3]Beijing Synchrotron Radiation Facility, Institute of High Energy Physics, Chinese Academy of Sciences, Beijing 100049, China

[4]GeoScience Department, Stony Brook University, Stony Brook, New York 11794, United States

[*]Correspondence and requests for materials should be addressed to B.B.L. (liubb@jlu.edu.cn)

Supplementary Figures

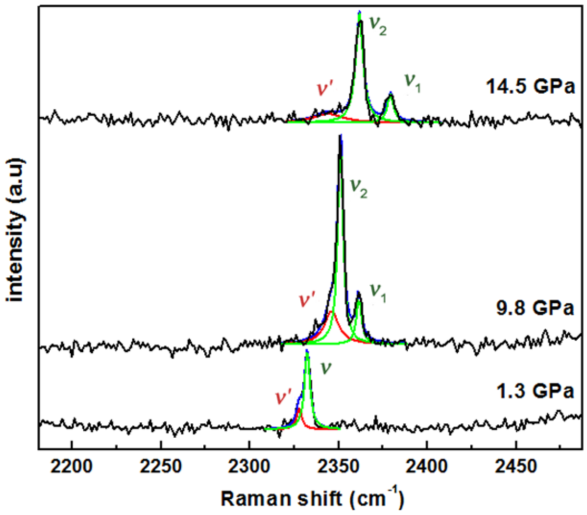

Figure S1. Raman spectra of nitrogen in the confined system at 1.3 GPa, 9.8 GPa and 14.5 GPa, the fitted curves show the ν', ν, ν_1 and ν_2 peaks.

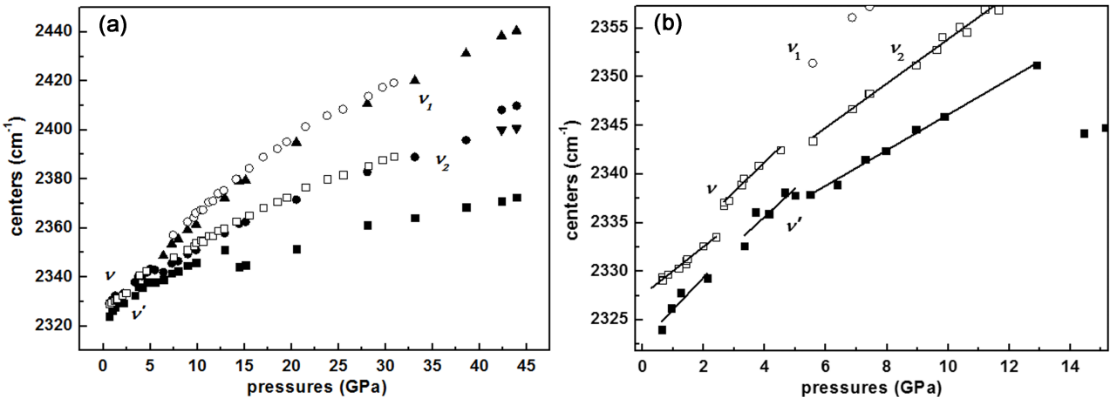

Figure S2. Pressure dependence of Raman spectra of nitrogen: open symbols are for the bulk nitrogen and closed symbols are for the nitrogen in the confined system. The full lines in (b) show the linear fits of v and v' in the low-pressure area. At pressure below 5.4 GPa, the vibrational mode v of nitrogen in the confined system and bulk nitrogen have similar peak positions and slope. At pressure above 5.4 GPa, the pressure dependences of the v_1 and v_2 vibrations are close to those of bulk nitrogen. This suggests that the v, v_1 and v_2 vibrations probably originate in the nitrogen outside the AFI channels.

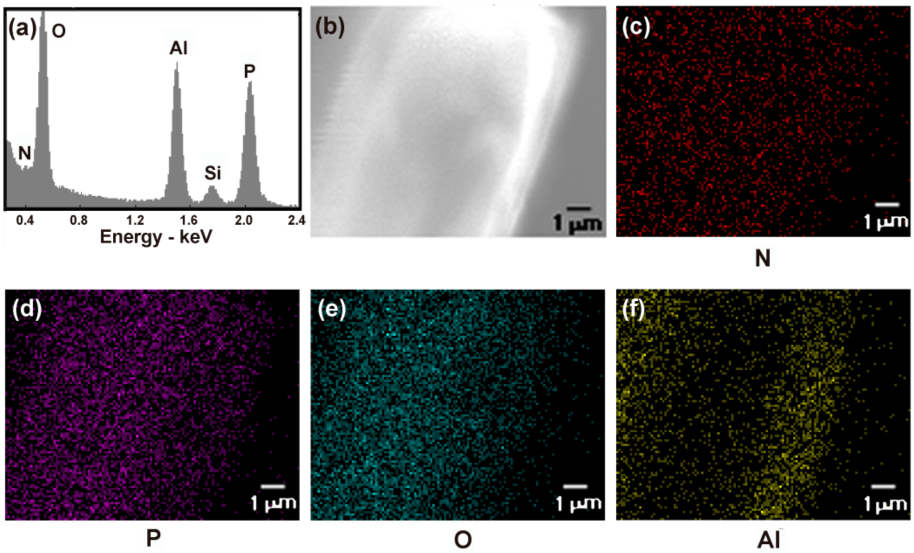

Figure S3. (a) EDX spectrum, (b) SEM image and (c-f) N, P, O and Al elemental mapping of the sample: nitrogen confined inside the AFI channels after releasing the pressure to 0 GPa.

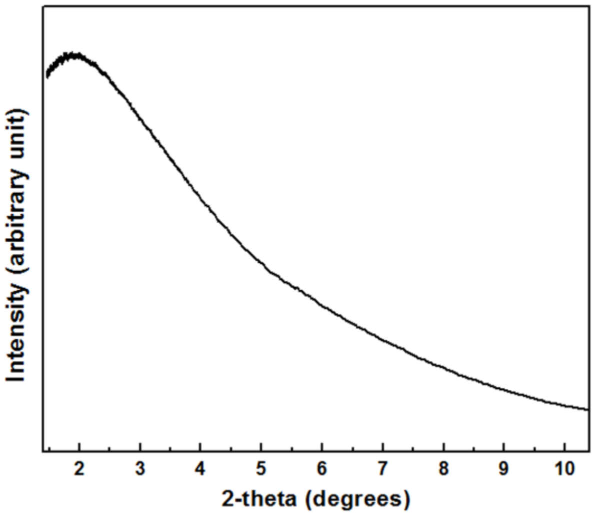

Figure S4. XRD pattern of the intact AFI released from high pressure. (λ = 0.40617 Å)

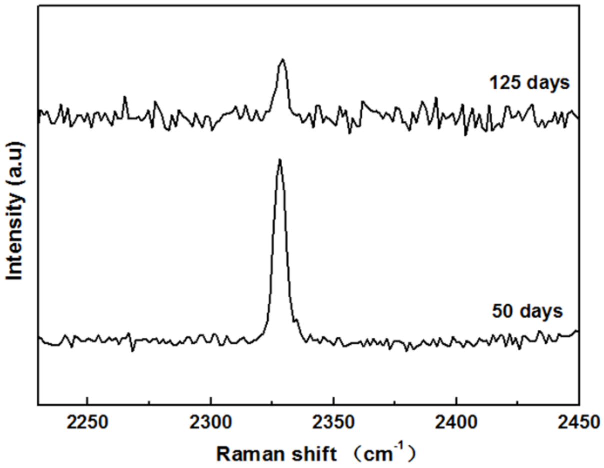

Figure S5. Raman spectra of nitrogen confined in the intact AFI sample after preservation for a long time at atmosphere pressure and room temperature. The Raman signal at about 2328.5 cm^{-1} is maintained for at least 125 days. The intensity of the signal decreases with an increasing length of the preservation period.
